# Identification and Partial Characterization of an L-Tyrosine Aminotransferase (TAT) from *Arabidopsis thaliana*

**DOI:** 10.1155/2010/549572

**Published:** 2010-08-04

**Authors:** Pranav R. Prabhu, André O. Hudson

**Affiliations:** School of Biological and Medical Sciences, Rochester Institute of Technology, 85 Lomb Memorial Drive, Rochester, NY 14623-5603, USA

## Abstract

The aminotransferase gene family in the model plant *Arabidopsis thaliana* consists of 44 genes. Twenty six of these enzymes are classified as characterized meaning that the reaction(s) that the enzyme catalyzes are documented using experimental means. The remaining 18 enzymes are uncharacterized and are therefore deemed putative. Our laboratory is interested in elucidating the function(s) of the remaining putative aminotransferase enzymes. To this end, we have identified and partially characterized an aminotransferase (TAT) enzyme from Arabidopsis annotated by the locus tag At5g36160. The full-length cDNA was cloned and the purified recombinant enzyme was characterized using *in vitro* and *in vivo* experiments. *In vitro* analysis showed that the enzyme is capable of interconverting L-Tyrosine and 4-hydroxyphenylpyruvate, and L-Phenylalanine and phenylpyruvate. *In vivo* analysis by functional complementation showed that the gene was able to complement an *E. coli* with a background of aminotransferase mutations that confers auxotrophy for L-Tyrosine and L-Phenylalanine.

## 1. Introduction

Aminotransferases (EC 2.6.1.x) are ubiquitous enzymes that catalyze the reversible interconversion of amino acids and their 2-oxoacid cognates. An amino acid usually serves as the amino donor while a 2-oxoacid serves as the amino acceptor. Aminotransferases are involved in a number of metabolic pathways including the anabolism and catabolism of amino acids, vitamin biosynthesis, carbon and nitrogen assimilation, secondary metabolism, and gluconeogenesis among others [1]. The cofactor pyridoxal-5′-phosphate (PLP) is necessary for aminotransferase activity. Pyridoxal-5′-phosphate is covalently bound to the enzyme through an imine linkage to the *ε*-amino group of conserved lysine residue in the active site of the enzyme [2]. All aminotransferases employ a ping-pong (double displacement) mechanism of action. In the first step, the amino acid substrate is converted to a 2-oxoacid cognate and in the second step; the 2-oxoacid substrate is converted to its cognate amino acid.

In the model organism *Arabidopsis thaliana*, there are 44 annotated aminotransferases and it was reported that only 25 of the 44 annotated aminotransferase enzymes have been characterized, meaning that the reactions catalyzed by the enzymes have been shown through experimental means [2]. The remaining 19 enzymes are classified as uncharacterized and as such are deemed putative. Recently, one of the uncharacterized enzymes annotated by the locus tag At4g33680 was characterized and was shown to be a *L,L*-diaminopimelate aminotransferase (EC 2.6.1.83) involved in a variant lysine biosynthetic pathway in plants and bacteria [3–5].

In Arabidopsis, there are 7 annotated tyrosine aminotransferases (TATs) (EC 2.6.1.5) which represent 16% of all aminotransferases. Out of the 7 predicted TATs from Arabidopsis, only two of them have been identified as having TAT activity: At4g23600 [6] and At2g20610 [7]. 

**Figure 1:**
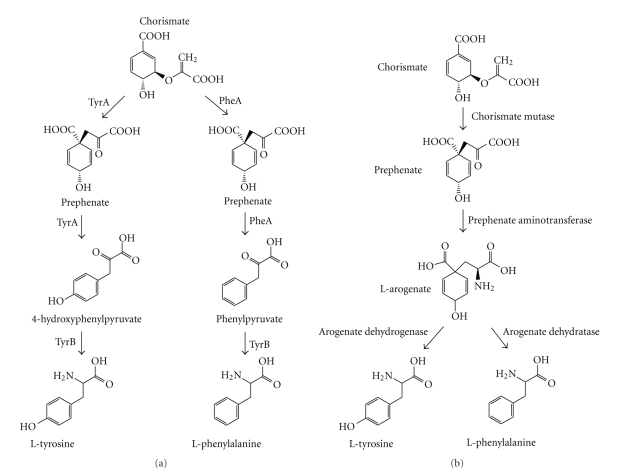
Pathways for L-Tyrosine and L-Phenylalanine biosynthesis in *E. coli* and plants. (a) shows the pathway for both amino acids from the intermediate chorismate in *E. coli*. Abbreviations in the diagram include PheA (chorismate mutase/prephenate dehydratase), TyrA (chorismate mutase, prephenate dehydrogenase), and TyrB (tyrosine aminotransferase). (b) depicts the plant pathway for the synthesis of the amino acids from the common intermediate L-Arogenate.

In *E. coli*, the aromatic amino acids L-Tyrosine, L-Phenylalanine and L-Tryptophan are all synthesized from the common intermediate chorismate (Figure 1(a)). The bifunctional enzyme, chorismate mutase (EC 5.4.99.5)/prephenate dehydratase (EC 4.2.1.51) encoded by the *pheA* gene catalyzes the conversion of chorismate to prephenate and prephenate to phenylpyruvate. For L-Tyrosine biosynthesis, the bifunctional enzyme chorismate mutase/prephenate dehydrogenase (EC 5.4.99.5) encoded by the *tyrA* gene catalyzes the conversion of chorismate to 4-hydroxyphenylpyruvate. The enzyme TAT encoded by the *tyrB* gene catalyzes the ultimate step in L-Tyrosine and L-Phenylalanine biosynthesis by the conversion of 4-hydroxyphenylpyruvate and phenylpyruvate to L-Tyrosine and L-Phenylalanine, respectively (Figure 1(a)). It should be noted that the aspartate aminotransferase (AspC) and the branched-chain aminotransferase (IlvE) are also involved in L-Tyrosine and L-Phenylalanine biosynthesis due to the fact that these enzymes have overlapping activities with TyrB from *E. coli* and *P. aeruginosa* [8–10].

In plants, the anabolism of L-Tyrosine and L-Phenylalanine biosynthesis proceeds through the intermediate chorismate. However, the pathway is distinctive from fungi and enteric bacteria in that plants have the amino transfer step at the penultimate step rather than the ultimate step. Chorismate is converted to prephenate by the enzyme chorismate mutase (EC 5.4.99.5) followed by a glutamate-dependent amino transfer reaction of prephenate catalyzed by prephenate aminotransferase (EC 2.6.1.79). L-Arogenate, the result of the amino transfer reaction, is either oxidized by the oxidoreductase enzyme arogenate dehydrogenase (EC 1.3.1.43) to form L-Tyrosine or converted to L-Phenylalanine by the lyase enzyme arogenate dehydratase (EC 4.2.1.91) (Figure 1(b)) [11–14].

Plants are known to synthesize the three aromatic amino acids within the plastids [15]. However, a cytosolic chorismate mutase has been identified in Opium poppy [16], Tobacco [17], and Arabidopsis [18]. Although the presence of a cytosolic postchorismate portion of the pathway is still debated, a recent report showed that the key enzymes, arogenate dehydrogenase, and arogenate dehydratase, involved in the synthesis of both L-Tyrosine and L-Phenylalanine, are localized within the plastids of Arabidopsis [19]. 

Here we present the identification and characterization of a TAT from Arabidopsis annotated by the locus tag At5g36160 that is deemed putative in the public databases. The cDNA was cloned and the purified recombinant enzyme was characterized by performing *in vivo* and *in vitro* experiments. Functional complementation analysis demonstrated that the gene is able to complement an *E. coli tyrB* mutant that confers auxotrophy for both L-Tyrosine and L-Phenylalanine. *In vitro* analysis showed that the enzyme was able to interconvert L-Tyrosine and 4-hydroxyphenylpyruvate and L-Phenylalanine and phenylpyruvate.

## 2. Material and Methods

### 2.1. Plant Growth and Conditions

*Arabidopsis thaliana* Col 7 Arabidopsis Biological Resource Center (ABRC) was grown on Murashige and Skoog (MS) medium in a growth chamber with a 16-hour light and an 8-hour dark period. The temperature was 24°C during the light period and 20°C during the dark. The light intensity was approximately 120 *μ*E M^−2^ sec^−1^.

### 2.2. RNA Isolation from Arabidopsis thaliana

Total RNA was isolated from 7 day old Col 7 Arabidopsis seedlings using TriZol reagent (Life Technologies). One hundred milligrams of seedlings was ground in liquid nitrogen and homogenized in 1 mL of TriZol, followed by incubation at room temperature for two minutes. Total RNA was precipitated using 1 mL 100% (v/v) isopropanol. The RNA pellet was washed three times with 1 mL 75% (v/v) ethanol. The RNA pellet was air-dried and resuspended in 30 *μ*L of Diethyl Pyrocarbonate- (DEPC-) treated water.

### 2.3. cDNA Synthesis

Total RNA isolated from Col 7 Arabidopsis seedlings was used to synthesize cDNA. The reaction contained 1 *μ*L oligo (dT)_12–18_ primer, 1 *μ*g total RNA, 1 *μ*L of 10 mM dNTP mix, and DEPC-treated water up to 13 *μ*L. The mixture was incubated at 65°C for 5 minutes, followed by incubation on ice for 5 minutes. The Reverse Transcription System Kit (Promega) was used to synthesize cDNA following the manufacturer's protocol.

### 2.4. Amplification and Cloning of the At5g36160 cDNA

The full-length cDNA of At5g36160 was amplified by PCR. The cDNA was amplified using twelve picomoles of forward and reverse primers, 1 mM MgSO_4_, 0.5 mM of each of the four deoxynucleotide triphosphates, 2 *μ*L of cDNA product, and 1 unit of Platinum *Pfx* DNA polymerase (Invitrogen) using the following PCR conditions: 1 cycle at 94°C for 2 minutes, followed by 36 cycles of 94°C for 15 seconds, 60°C for 30 seconds, and 72°C for 2 minutes. The forward and reverse primers used to amplify the gene were 5′-CCCCGAATTC ***ATG***GGAGAAAACGGAGCCAAGCGAT-3′ and 5′-CCCCGAGCTC ***TCA***TGGCTGATTCTTGGAGTGTCTC-3′.

The underlined sequence represents the restriction enzyme sites used in the cloning of the cDNA while the bolded and italicized sequences represent initiation and termination codons. For cloning, the PCR fragment was digested with *EcoRI* and *SacI *and ligated into the plasmid pET30a (Novagen) to produce the plasmid pET30a + At5g36160. The recombinant protein derived from this plasmid carries a hexahistidine and S-TAG epitope derived from pET30a plasmid at the amino terminus. To confirm the fidelity of the PCR reaction, the PCR fragment was sequenced from the pET30a construct using the T7 promoter primer 5′-TAATACGACTCACTATAGGG-3′ and the T7 terminator primer 5′-TATGCTAGTTATTGCTCAG-3′, and was compared to the published sequence deposited in the public database (http://www.arabidopsis.org/).

### 2.5. Functional Complementation Plasmid Construct

The plasmid used for functional complementation of the *E. coli* DL39 mutant was produced by subcloning the *XbaI* and *HindIII *fragment from pET30a + At5g36160 into pBAD33 [20], to produce pBAD33 + At5g36160. The fusion protein produced from the pBAD33 construct is identical to the protein produced from the pET30a construct.

### 2.6. Functional Complementation

The *E. coli* auxotrophic mutant DL39 was obtained from the Coli Genome Stock Center (CGSC) located at Yale University (http://cgsc.biology.yale.edu/). The DL39 strain (no. 6913) has the following genotype (*LAM-, aspC13, fnr-25, rph-1 ilvE12, tyrB507*) [21]. For functional complementation, the strain was transformed with either pBAD33 or pBAD33 + At5g36160. Colonies were selected on Luria-Bertani (LB) agar plates supplemented with 50 *μ*g mL^−1^ L-Tyrosine, 50 *μ*g mL^−1^ L-Phenylalanine, and 34 *μ*g mL^−1^ chloramphenicol. Fifty individual colonies were then replicated onto M9 agar plates supplemented with 50 *μ*g mL^−1^ L-Phenylalanine and 50 *μ*g mL^−1^ L-Tyrosine, 0.5% (w/v) glycerol, 0.2% (w/v) arabinose, 50 *μ*g mL^−1^ L-Aspartate, 50 *μ*g mL^−1^ L-Leucine, 50 *μ*g mL^−1^ L-Valine, 50 *μ*g mL^−1^ L-Isoleucine, 10 *μ*g mL^−1^ uracil and also on plates lacking L-Phenylalanine and L-Tyrosine. To test whether the cDNA was able to complement the L-Aspartate and Branched-chain amino acids auxotrophy in DL39, replica-plating was carried out on M9 media supplemented with or without the amino acid(s). The strains were incubated at 30°C for 48 hours. The growth curve of DL39 expressing the pBAD33 or pBAD33 + At5g36160 plasmid was carried out by inoculating 50 mL M9 media supplemented with 0.5% (w/v) glycerol, 0.2% (w/v) arabinose, 50 *μ*g mL^−1^ L-Aspartate, 50 *μ*g mL^−1^ L-Leucine, 50 *μ*g mL^−1^ L-Valine, 50 *μ*g mL^−1^ L-Isoleucine, and 10 *μ*g mL^−1^ uracil with 1 mL of overnight culture. The absorbance at 600 nm was documented at one hour intervals up to 12 hours.

### 2.7. Protein Expression and Purification

The plasmid pET30a + At5g36160 was transformed into *E. coli *BL21-CodonPlus-RIPL strain (Stratagene). For protein expression and purification, the strain was grown in 1.0 L LB broth containing 50 *μ*g mL^−1^ kanamycin and 34 *μ*g mL^−1^ chloramphenicol at 37°C to an OD_600 nm_ of 0.5. Protein expression was induced by adding isopropyl *β*-D-1-thiogalactopyranoside (IPTG) to a final concentration of 0.2 mM at 25°C for 5 hours. For purification, the cells were lysed by sonication in a solution of 50 mM sodium phosphate and 300 mM NaCl (pH 8.0). The soluble protein extract was incubated with 1.0 mL bed volume of Talon Metal Affinity Resin (Clontech) for 30 minutes at 4°C. The resin was washed with 300 mL of sonication buffer containing 10 mM imidazole (pH 8.0), and the enzyme was eluted using 50 mL of sonication buffer containing 250 mM imidazole (pH 8.0). The purified enzyme was concentrated using an Amicon Ultra 10,000 molecular weight cutoff filter device, replacing the elution buffer with 100 mM HEPES-NaOH containing 1 mM DTT and 1 mM EDTA (pH 8.2). For long-term storage, the enzyme was stored in 50% (v/v) glycerol. Protein concentration was measured using the Bradford assay with bovine serum albumin as the standard [22].

### 2.8. Enzyme Assays

Two different enzyme assays were used to measure TAT activity. The forward assay measured the production of phenylpyruvate and 4-hydroxphenylpyruvate from L-Phenylalanine and L-Tyrosine degradation, respectively. The reverse assay measured the production of 2-ketoglutarate from the transamination of phenylpyruvate or 4-hydroxyphenylpyruvate using the coupling enzyme 2-ketoglutarate dehydrogenase. For the forward reaction, the standard assay consisted of 10 mM donor (L-Tyrosine or L-Phenylalanine), 10 mM acceptor (2-ketoglutarate, pyruvate, oxaloacetate, oxovalerate, prephenate), and pure recombinant At5g36160 in 100 mM HEPES-NaOH (pH 8.2) buffer in a final volume of 0.4 mL. The reactions were incubated at 30°C for 1 hour and 0.1 mL of 2 N NaOH was added to stop the reaction. The production of phenylpyruvate and 4-hydroxyphenylpyruvate was measured at *A*_320_ and *A*_331_, respectively, using the extinction coefficient of 17,500 M^−1^ for phenylpyruvate and 32,850 M^−1^ for 4-hydroxyphenylpyruvate [23]. For the reverse reaction, the standard assay consisted of 0.3 mM NAD, 5 mM glutamate, 10 mM phenylpyruvate or 4-hydroxyphenylpyruvate, 0.3 mM CoA, 200 *μ*g 2-ketoglutarate dehydrogenase (0.85 *μ*mol min^−1^ mg^−1^ protein) (Sigma), and pure recombinant At5g36160 in 100 mM HEPES-NaOH (pH 8.2), in a final volume of 0.5 mL. The production of NADH was measured at *A*_340_. Both the forward and reverse assays were incubated at 30°C. All kinetic constants were calculated using GraphPad Prism V3.03 software by using the nonlinear regression analysis algorithm feature. A Beckman DU640 Spectrophotometer was used to monitor enzyme activity.

### 2.9. Multiple-Protein Sequence Alignment and Phylogenetic Tree Construction

TAT protein sequences from Arabidopsis were aligned using the program MegAlign (DNAstar) using the ClustalW algorithm. A boxshade representation of the alignment was produced using the Chromatic Representation Of Multiple Alignments (CHROMA) freeware [24]. The accession numbers for the Arabidopsis TATs are presented in Table 1. The phylogenetic tree was constructed by creating a protein alignment using ClustalW and the tree was drawn using the TREEVIEW program [25]. The accession numbers for the bacterial and mammalian enzymes are presented in the tree adjacent to the abbreviated initials for the genus and species of each bacterial TAT (Ec: *Escherichia coli*, Pa: *Pseudomonas aeruginosa*, Bp: *Bordetella petrii*, and Se: *Salmonella enterica*) and mammalian TAT (Dr: *Danio rerio*, Xt: *Xenopus tropicalis,* Hs: *Homo sapien*, Mm: *Mus musculus*, and Rn: *Rattus norvegicus*).

## 3. Results and Discussion

### 3.1. The Tyrosine Aminotransferase Gene Family in Arabidopsis thaliana

**Figure 2:**
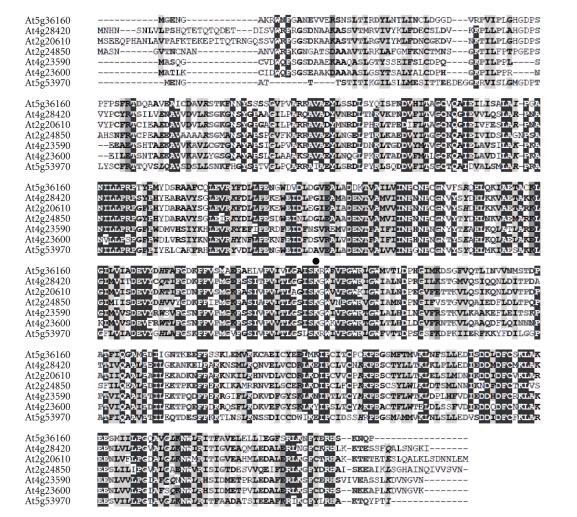
Amino acid sequence alignment of the TAT family from Arabidopsis. The method used for creating the alignment is described in Section 2. The black circle denotes the PLP ligand at the conserved lysine residue.

There are 44 annotated aminotransferases in *Arabidopsis thaliana* [1]. Seven out of the reported 44 enzymes are predicted to encode TATs. The accession numbers, locus tags, and updated annotated information of the TAT are presented in Table 1. Also presented is the information of the *E. coli* ortholog annotated by the accession number NP_418478. A comparative analysis as it pertains to the amino acid sequence of the enzymes showed that the *E. coli* ortholog is only 7.6% identical throughout the entire protein sequence when compared to the plant enzyme that is the focus of this study (Table 1). The protein sequence alignment of the seven TATs from Arabidopsis shows that they are fairly conserved (Figure 2). The percent identity ranges from 54.1% to 38.8% when the other TAT proteins are compared to At5g36160 (Table 1).

### 3.2. The Gene Annotated by the Locus Tag At5g36160 Encodes TAT

**Figure 3:**
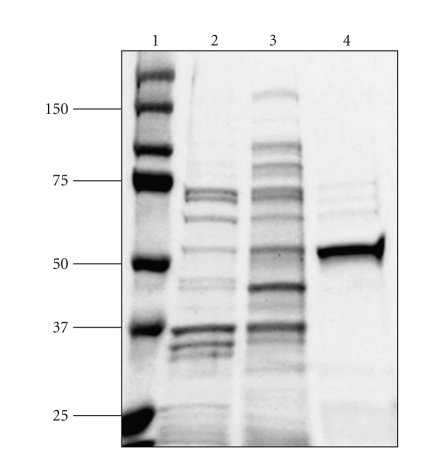
Recombinant expression and purification of At5g36160 from the *E. coli* strain BL21 Codon Plus RIPL. The enzyme was purified as described in Section 2. Along with the molecular mass markers (kDa) (lane 1), the gel shows the profile of 10 *μ*g soluble proteins extracted from uninduced (lane 2) and induced cells (lane 3). Lane 4 shows the profile of 1.0 *μ*g of the purified recombinant enzyme. The proteins were resolved on an SDS-PAGE gel containing 10% (w/v) acrylamide, and the gel was stained using Coomassie Blue.

**Figure 4:**
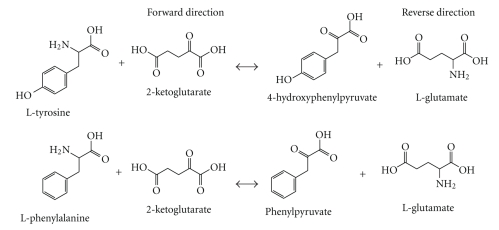
Reactions catalyzed by TATs. Reactions catalyzed by the enzyme in the forward and reverse directions. In the forward direction, L-Tyrosine and L-Phenylalanine serve as the amino donors while 2-ketoglutarate serves as the acceptor. In the reverse direction, glutamate serves as the amino donor while 4-hyrdroxyphenylpyruvate and phenylpyruvate serve as the acceptors.

**Figure 5:**
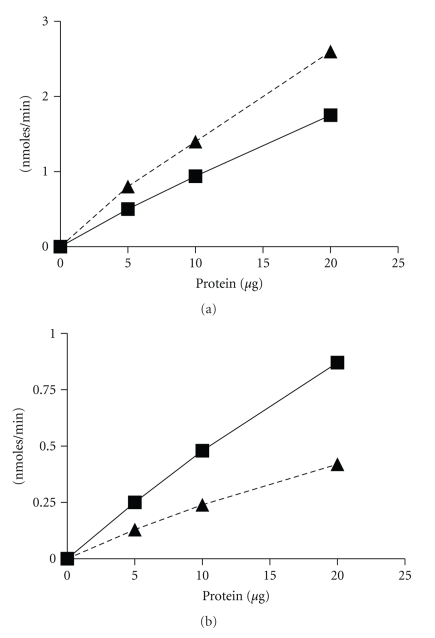
Detection of TAT activity in the forward and reverse directions. Both graphs show the correlation between reaction rate and the amount of enzyme added to the reaction. (a) shows the synthesis of 4-hydroxyphenylpyruvate (square) and phenylpyruvate (triangle) in the forward direction, while (b) shows the synthesis of 2-ketoglutarate synthesized from 4-hyrdoxyphenylpyruvate (square) and phenylpyruvate (triangle) in the reverse direction. The standard assays used for this analysis are described in Section 2.

To assess the function of At5g36160, the cDNA was cloned and the enzyme was purified using affinity chromatography (Figure 3). The pure recombinant protein was used to perform enzyme assays to answer the question of whether the enzyme is capable of catabolizing L-Tyrosine and L-Phenylalanine to either 4-hydroxyphenylpyruvate or phenylpyruvate, respectively. Since most aminotransferases are able to catalyze reversible reactions, the enzyme should also be able to synthesize L-Tyrosine and L-Phenylalanine from 4-hydroxyphenylpyruvatre and phenylpyruvate, respectively, given the proper amino donor. In the forward direction, the substrates L-Tyrosine and L-Phenylalanine are the amino donors while 2-ketoglutarate is the amino acceptor. In the reverse direction, glutamate serves as the amino donor while 4-hydroxyphenylpyruvate and phenylpyruvate serve as the amino acceptors (Figure 4).  Figure 5(a) shows the synthesis of 4-hydroxyphenylpyruvate and phenylpyruvate from the transamination of L-Tyrosine and L-Phenylalanine when increasing amounts of enzyme was added to the assay. The correlation observed for the synthesis of 4-hydroxyphenylpyruvate and phenylpyruvate is consistent with the synthesis of 2-ketoglutarate, which is a coproduct formed by the transamination of these substrates (Figure 5(b)). No enzymatic activity was observed when either the amino donors or the amino acceptors were omitted in the forward and reverse assays (data not shown). Moreover, a mock purification was done using a plasmid only (pET30a) control using the same purification scheme. Enzyme activity was not observed when using the eluate from the mock purification, showing that the activity observed in the forward and reverse assay is from the recombinant enzyme and not from the native *E. coli *BL21-CodonPlus-RIPL TAT enzyme.

### 3.3. The Gene Annotated by the Locus Tag At5g36160 Is Able to Functionally Complement the E. coli L-Tyrosine and L-Phenylalanine Auxotrophic Mutant DL39

**Figure 6:**
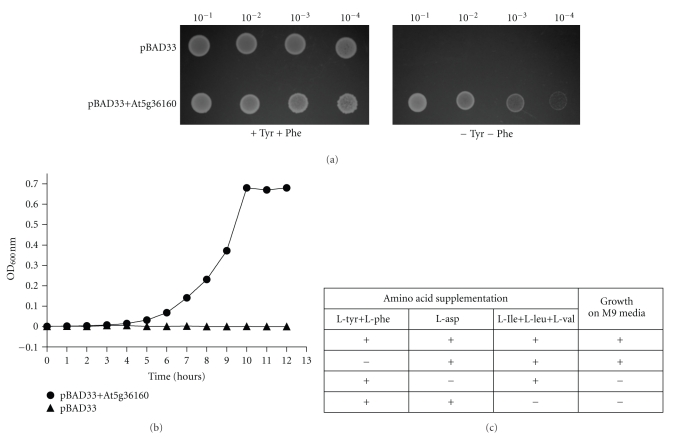
(a) Functional complementation assay. Functional complementation was tested using the *E. coli *mutant DL39. The plasmids used were pBAD33 and pBAD33 + At5g36160. The bacteria were grown to an OD of 1.0 measured at 600 nm, the strains were serially diluted to 10^−1^, 10^−2^, 10^−3^, and 10^−4^ using 0.85% (w/v) saline, and 5 *μ*L was replica plated on medium with or without L-Tyrosine and L-Phenylalanine supplemented with 0.2% (w/v) arabinose as described in Section 2. It should be noted that 50 individual transformants were replica plated and tested using the complementation assay. All 50 bacteria transformed with the empty vector were unable to grow on media lacking L-Tyrosine or L-Phenylalanine while all 50 transformants expressing the plant enzyme were able to grow on media lacking L-Tyrosine and L-Phenylalanine. For presentation purposes, only one control and one experimental transformant were used in the diagram. (b) shows the growth curve of the DL39 strain transformed with either pBAD33 (triangle) or pBAD33 + At5g36160 (circle) in M9 medium lacking L-Tyrosine and L-Phenylalanine supplemented with 0.2% (w/v) arabinose. (c) shows the summary of the functional complementation assay of the plant enzyme ability to complement the *E. coli* triple mutant on M9 medium.

**Figure 7:**
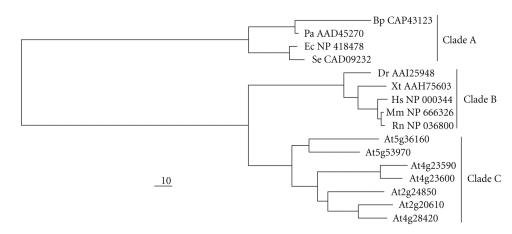
Phylogenetic tree depicting the evolutionary relationship between TATs from bacteria (Clade A), mammals (Clade B), and Arabidopsis (Clade C). The protein sequences were aligned using the ClustalW and the neighbor joining tree was constructed using the TREEVIEW software.

Given the fact that the pure recombinant enzyme was able to synthesize L-Tyrosine and L-Phenylalanine *in vitro*, it should be possible to complement an *E. coli* mutant devoid in TAT activity. To confirm this, a functional complementation assay was used because it was previously reported that a TAT from *Pseudomonas aeruginosa* (*phyC*) was able to complement the DL39 *E. coli* mutant [26]. DL39 is a triple mutant that has mutations in the aminotransferase genes *aspC* (L-Aspartate aminotransferase), *tyrB* (L-Tyrosine aminotransferase), and *ilvE *(branched-chain aminotransferase) which have been shown to have overlapping activities in *E. coli *[26]. As a result of these mutations, the bacterium is auxotrophic for the amino acids L-Aspartate, L-Tyrosine, L-Phenylalanine, and the branched-chain amino acids: L-Leucine, L-Valine, and L-Isoleucine. The DL39 strain was transformed with either an empty plasmid or a plasmid expressing the At5g36160 protein. While the mutant is able to grow on media supplemented with L-Tyrosine and L-Phenylalanine, only the mutant strain expressing the plant enzyme is able to grow on L-Tyrosine and L-Phenylalanine free media (Figure 6(a)). Figure 6(b) shows the graph corresponding to growth curve of the *E. coli* mutant transformed with the empty plasmid or the plasmid expressing the plant TAT enzyme. Since aminotransferases are known to be promiscuous enzymes, and given the fact that the DL39 *E. coli* mutant contains a mutation in the branched-chain aminotransferase gene, the growth on media containing L-Tyrosine, L-Phenylalanine, and L-Aspartate but lacking the amino acids L-Leucine, L-Valine, and L-Isoleucine was tested. The results from this analysis showed that the plant enzyme was not able to functionally complement the branched-chain amino acid auxotrophy in the DL39 *E. coli* mutant (Figure 6(c)). The DL39 *E. coli* mutant also harbors a mutation in the aspartate aminotransferase gene (*aspC*); the plant enzyme was not able to complement the L-Aspartate auxotrophy using the functional complementation assay (Figure 6(c)). These results indicate that the plant enzyme annotated by the locus tag At5g36160 encodes TAT and it is not involved in the anabolic pathways for L-Leucine, L-Valine, L-Isoleucine, and L-Aspartate. Although the plant enzyme is only 7.6% identical to the *E. coli* TAT enzyme, it was able to complement the *E. coli* mutant. In fact, based on a phylogenetic tree comparing sequence similarity, the plant TATs, bacterial TATs and the mammalian TATs are divergent even though the enzymes are grouped in the same subdivision within the same subgroup of the class I aminotransferases [2, 27, 28]. The sequence similarity between the three groups of TATs is shown in a phylogenetic tree which depicts three distinct clades. Clade A represents the bacterial enzymes from several bacteria; clade B represents a selection of mammalian enzymes, and clade C represents the Arabidopsis TAT enzyme family (Figure 7).

### 3.4. Physical Properties of At5g36160: Effects of pH, Temperature on Enzyme Activity

The transamination reaction using the forward assay with L-Tyrosine as the donor and 2-ketoglutarate as the acceptor was carried out at various temperatures and various pH levels. The enzyme had an optimal activity between the temperature range of 30–40°C. The enzyme lost half of its activity at 60°C and was inactive at 90°C (see Figure 1(a) in Supplementary Material available online at doi:10.1155/2010/549572). The activity of the pure enzyme was examined using the buffers HEPES-NaOH ranging in pH values from 6.8–8.2 (Supplementary Figure 1(b)) and TRIS-HCL ranging in values from 7.0–9.0 (Supplementary Figure 1(c)). Maximum activity was observed when HEPES-NaOH (pH 8.2) was used.

### 3.5. Subunit Structure of At5g36160

The subunit structure of most aminotransferases is dimeric in nature containing two identical subunits. The molecular weight of aminotransferases in plants ranges between 48,000 Daltons for ornithine aminotransferase in squash [29], to 100,000 Daltons for soybean aspartate aminotransferase [30]. The molecular weight of the pure recombinant TAT was estimated using SDS-PAGE analysis and found to be approximately 52 kDa (Figure 3). If the holoenzyme is active as a dimer, the recombinant At5g36160 is approximately 100 kDa which is consistent with other plant aminotransferases [3]. Moreover, the structure of the *E. coli* TyrB was solved by X-ray crystallography and the holoenzyme was shown to be homodimeric [31]. It should be noted that we attempted to use size exclusion HPLC to gather information regarding the subunit structure of the recombinant enzyme but the results were not consistent with the SDS-PAGE results due to the multiple oligomerization states of the purified recombinant enzyme. Future experiments using gel filtration chromatography of the native enzyme would be helpful in determining the actual subunit structure of this enzyme.

### 3.6. Absorption Spectrum Analysis of At5g36160

**Figure 8:**
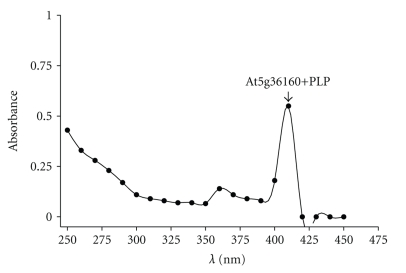
Absorption spectrum of At5g36160. UV-visible absorption spectrum of the native purified recombinant enzyme (50 *μ*g) in 100 mM HEPES-NaOH (pH 8.2). The PLP peak representing the PLP form of the enzyme is highlighted with an arrow.

Aminotransferases require PLP for activity because PLP acts as an electron sink, accepting electrons from substrates [32]. The purified At5g36160 was yellowish in color suggesting that PLP was bound to the enzyme. This was corroborated by UV-visible light wavelength scan analysis. The analysis revealed an absorption peak centered around 410 nm consistent with the presence of PLP along with a shoulder peak centered around 360 nm (Figure 8). It should be noted that free PLP has an absorption maximum centered around 390 nm. The detection of the PLP-form from the purified recombinant enzyme is indicative of a Schiff-base linkage with the *ε*-amino group of a conserved L-Lysine residue at position 252 in the amino acid sequence (Figure 2). This is consistent with other aminotransferase enzymes that have been characterized [3]. Moreover, a comparative protein alignment using At5g36160 and another aminotransferase At4g33680 that was solved by X-ray crystallography showed that the lysine at position 252 is the PLP ligand [33] denoted by the conserved PLP binding motif (data not shown).

### 3.7. Kinetic Properties of At5g36160

The kinetic properties of the enzyme was tested at several concentrations of one substrate and at the saturation levels of other substrates. The reciprocal plots for all 6 substrates were linear and were consistent with Michaelis-Menten kinetics. The *V*_max_ for the forward and reverse directions were calculated along with the apparent *K*_*m*_ for the various substrates. The enzyme has a maximum velocity of approximately 5.0 *μ*moles min^−1^ mg^−1^ in the forward direction and 23.5 *μ*moles min^−1^ mg^−1^ in the reverse direction. The apparent *K*_*m*_ for the substrates were 0.19 mM for L-Tyrosine, 0.84 mM for L-Phenylalanine, 1.2 mM for 2-ketoglutarate, 1.4 mM for L-glutamate, 0.22 mM for 4-hydroxyphenylpyruvate, and 0.13 mM for phenylpyruvate (Table 2(a)). The physical and kinetic properties of the enzyme are similar to the ones reported for the *E. coli* enzyme [8, 9]. The saturation plots along with the assay conditions that were used to calculate the *K*_*m*_ of the substrates using nonlinear regression analysis are provided as supplementary material (Figure 2).

### 3.8. Substrate Specificity

Aminotransferases are known to be promiscuous enzymes because conceptually, they catalyze the same reaction by transferring an amino group from a donor to an acceptor. We tested different acceptor compounds with the forward assay by using L-Tyrosine as the amino donor with 2-ketoglutarate, pyruvate, oxaloacetate, oxovalerate, or prephenate as the acceptors. The results from this analysis showed that the enzyme is not specific for 2-ketoglutarate but is able to use other amino acceptors as a substrate although 2-ketoglutarate is the preferred substrate (Table 2(b)). The ability of At5g36160 to use different substrates with regards to amino donors was not a part of this study.

### 3.9. The Role of TAT in Plants

**Figure 9:**
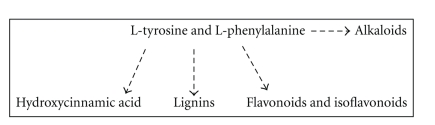
Secondary metabolites derived from the catabolism of L-Tyrosine and L-Phenylalanine in plants. The synthesis of secondary metabolites that are involved in plant defense occurs via the degradation of L-Tyrosine and L-Phenylalanine through the phenylpropanoid pathway.

The *de novo* synthesis of L-Tyrosine and L-Phenylalanine occurs within the plastids of plants [12, 16]. The subcellular localization of At5g36160 has not been demonstrated through experimentation. However, the enzyme is predicted to be localized in the cytosol by TargetP (http://www.cbs.dtu.dk/services/TargetP/) [34]. Plant TATs are more likely to be involved in the synthesis of secondary metabolites, given the fact that the enzyme is not necessary for L-Tyrosine and L-Phenylalanine synthesis in plants unless an unknown bacterial-like postchorismate pathway for the synthesis of L-Tyrosine and L-Phenylalanine exists in the cytoplasm of plants (Figures 1(a) and 1(b)). Moreover, secondary metabolites including alkaloids, lignins, flavonoids, isoflavonoids, and hydroxycinnamic acids are all derived from the phenylpropanoid catabolic pathway of L-Tyrosine and L-Phenylalanine (Figure 9) [35]. For example, 4-hydroxyphenylpyruvate is converted to homogentisate by the enzyme 4-hydoxyphenylpyruvate dioxygenase, which is the precursor compound for *α*-tocopherol and plastoquinones. Secondary metabolites are synthesized by plants in response to biotic and abiotic stresses and these compounds have roles relating to mechanical strength, pollen viability, pest deterrence, UV protection, and disease resistance among others [35]. An example of secondary metabolite synthesis in response to biotic stress has been studied in Arabidopsis. *Pseudomonas syringae *produces the phytotoxin coronatine. Coronatine belongs to the polyketide class of secondary metabolites and it is an analog of octadecanoids (jasmonic acid or 12-oxo-phytodienoic acid). Typically, a chlorotic phenotype is observed when the plant is attacked by certain pathovars of *P. syringae* that synthesize coronatine [36]. However, in response to the phytotoxin, Arabidopsis produces and accumulates anthocyanins derived from the phenylpropanoid pathway [37, 38]. The production of flavonoids is important because they were shown to possess antioxidant properties which are involved in protection of the plant cells from free radicals [39]. Since coronatine was shown to upregulate the mRNA expression of the TAT gene annotated by the locus tag At4g23600 (Table 1) [6], we suspect that At5g36160 might have a similar role in plants. The involvement of aminotransferases in the plant defense response is not a new phenomenon. Two aminotransferases, AGD2 annotated by the locus tag At4g33680 and ALD1 annotated by the locus tag At2g13810 have been shown to be integral in the biosynthesis of a compound(s) that is involved in phytopathogen resistance or tolerance [40, 41]. Also, expression of a glyoxylate aminotransferase in *Cucumis melo* was shown to confer protection against *Pseudoperonospora cubensis* which is the cause of downy mildew in wild melons [42]. Our study does not address the question of whether At5g36160 is involved in plant defense response, but we envision future studies will address this topic by creating transgenic plants that overexpress TAT to address the question of phytopathogen tolerance to the bacteria *P. syringae* and by studying the effect(s) that exogenous coronatine and other phytotoxins have on the mRNA levels of TAT genes.

## Supplementary Material

The supplementary data provided shows the data in graphical form for the physical and kinetic properties for the enzyme. Figure 1A shows the graph for the temperature optimum for the enzyme and Figure 1B and Figure 1C shows the pH optimum using two different buffers. Figure 2 depicts the saturation curves used to calculate the kinetic parameters of the enzyme. Along with the graphs, detailed descriptions of the assays are listed with respect to substrates amounts and enzyme concentration.

## Figures and Tables

**Table 1:** Locus tag and accession numbers for TATs from Arabidopsis and from *E. coli*. The accession numbers and predicted function for the enzymes were obtained from GenBank*™* (www.ncbi.nlm.nih.gov/Genbank/) and from The Arabidopsis Information Resource (TAIR) (www.arabidopsis.org/). The closed circle refers to the Arabidopsis plant enzyme that was actually studied and the asterisk denotes the accession number of the *E. coli *TAT enzyme.

Accession number	Locus tag	% identity to At5g36160	Annotation in GenBank*™*
NP_198465^•^	At5g36160	—	Putative tyrosine aminotransferase
AAN31921	At5g53970	54.1	Putative tyrosine aminotransferase
CAB79644	AT4g28420	48.3	Putative tyrosine aminotransferase
AAG37061	At2g20610	47.4	Putative tyrosine aminotransferase
AAP31937	At2g24850	44.8	Tyrosine aminotransferase
CAB79314	AT4g23590	39.0	Putative tyrosine aminotransferase
CAB79315	At4g23600	38.8	Putative tyrosine aminotransferase
NP_418478*	b4054	7.6	Tyrosine aminotransferase

**Table:** (a)

Assay	*V*_max _	*K*_cat_	Substrate	*K*_*m*_
Forward	5 ± 0.3	0.08	L-Tyrosine	0.19 ± 0.16
			L-Phenylalanine	0.84 ± 0.2
			2-ketoglutarate	1.2 ± 0.27

Reverse	23.5 ± 1.0	6.1	L-Glutamate	1.4 ± 0.16
			4-hydroxyphenylpyruvate	0.22 ± 0.05
			Phenylpyruvate	0.13 ± 0.06

**Table:** (b)

Acceptor/Donor combinations	Relative Activity (%)
L-Tyrosine/2-ketoglutarate	100
L-Tyrosine/oxaloacetate	75
L-Tyrosine/pyruvate	40
L-Tyrosine/prephenate	23
L-Tyrosine/oxovalerate	3
