# Supplementary material for: Identification and Partial Characterization of an L-Tyrosine Aminotransferase (TAT) from Arabidopsis thaliana
Source: Biochem Res Int. 2010 Aug 4;2010:549572. doi: 10.1155/2010/549572 (PMC3005984; doi:10.1155/2010/549572)
Supplement: Supplementary file 1 — The supplementary data provided shows the data in graphical form for the physical and kinetic properties for the enzyme. Figure 1A shows the graph for the temperature optimum for the enzyme and Figure 1B and Figure 1C shows the pH optimum using two different buffers. Figure 2 depicts the saturation curves used to calculate the kinetic parameters of the enzyme. Along with the graphs, detailed descriptions of the assays are listed with respect to substrates amounts and enzyme concentration. [file 549572.f1.pdf]

Supplementary Material

Figure. 1 Physical properties of At5g36160 effects of temperature and pH on enzyme activity. The transamination reaction was carried out using the forward assay using L-Tyrosine (10 mM) as the amino donor and 2-ketoglutarate (10 mM) as the amino acceptor by measuring the production of 4-hydroxyphenylpyruvate at 320 nm.

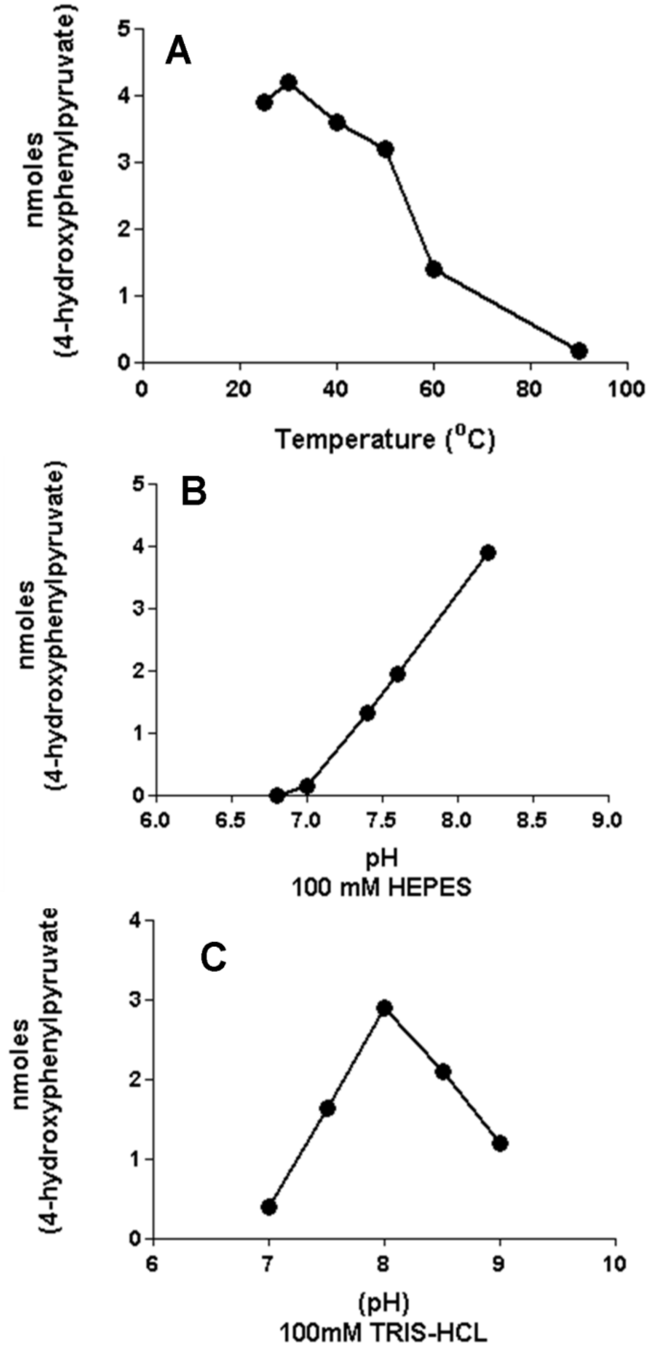

Figure. 2

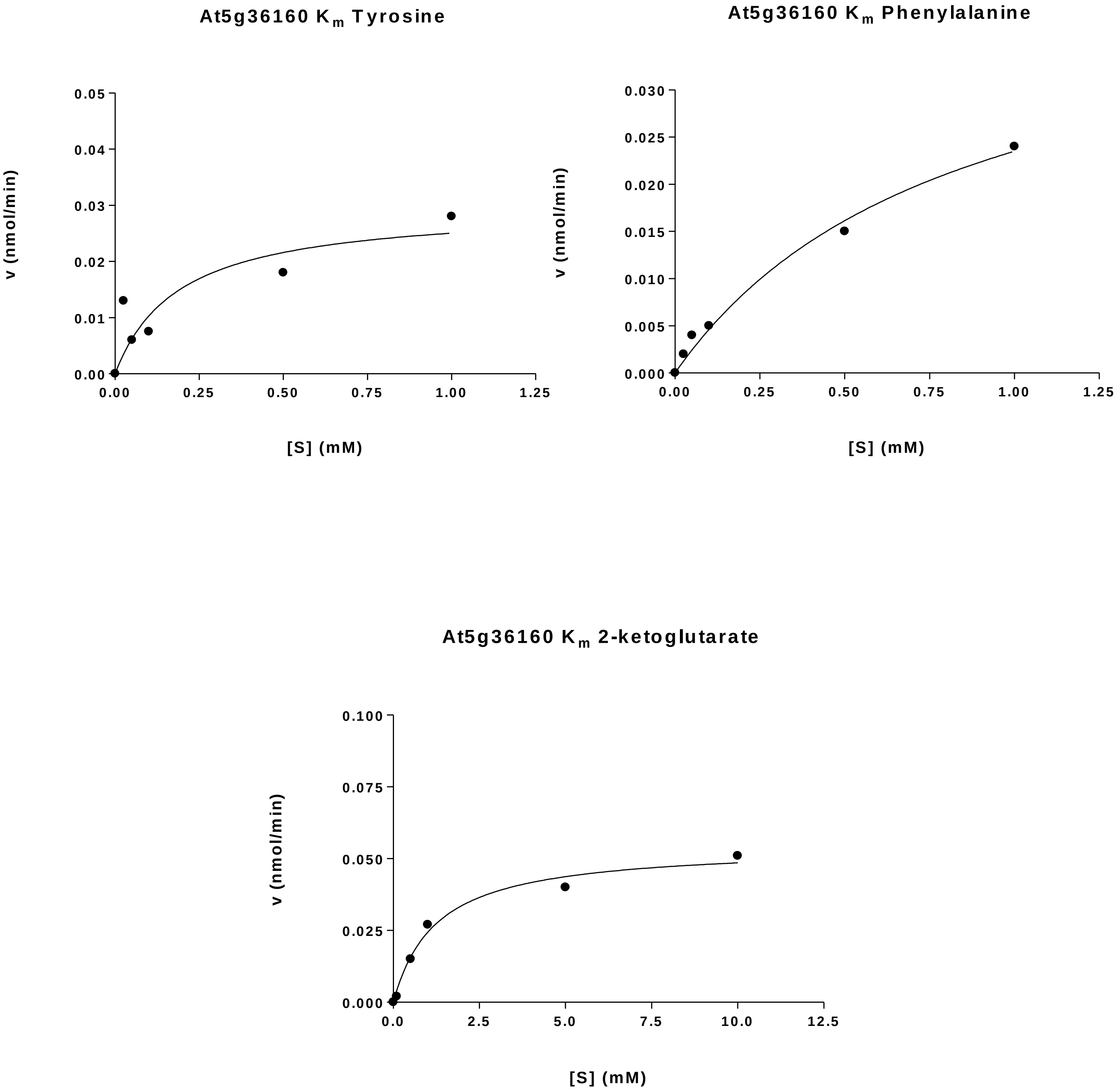
At5g36160 K$_m$ Tyrosine
v (nmol/min)
0.00
0.01
0.02
0.03
0.04
0.05
0.00
0.25
0.50
0.75
1.00
1.25
[S] (mM)
At5g36160 K$_m$ Phenylalanine
v (nmol/min)
0.000
0.005
0.010
0.015
0.020
0.025
0.030
0.00
0.25
0.50
0.75
1.00
1.25
[S] (mM)
At5g36160 K$_m$ 2-ketoglutarate
v (nmol/min)
0.000
0.025
0.050
0.075
0.100
0.0
2.5
5.0
7.5
10.0
12.5
[S] (mM)

At5g36160 K_m 4-hydroxyphenylpyruvate

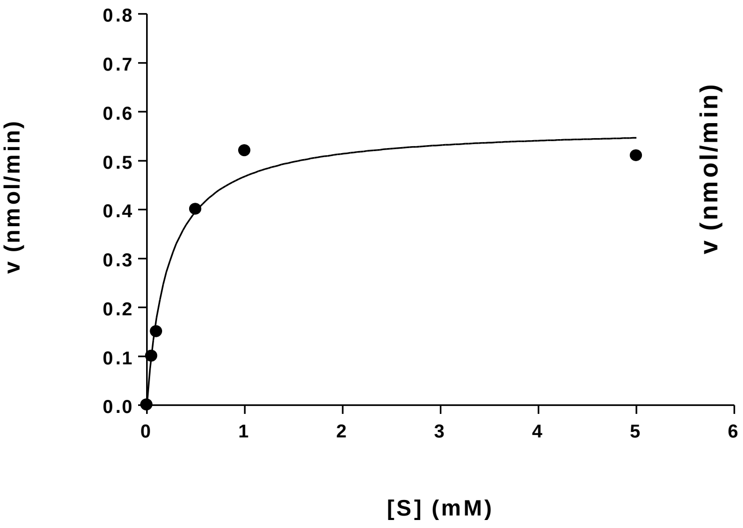

At5g36160 K_m Phenylpyruvate

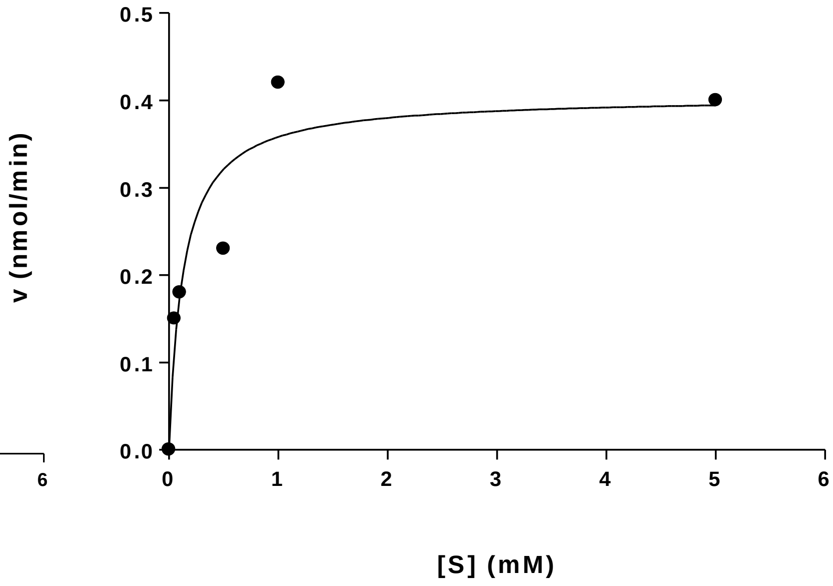

At5g36160 K_m Glutamate

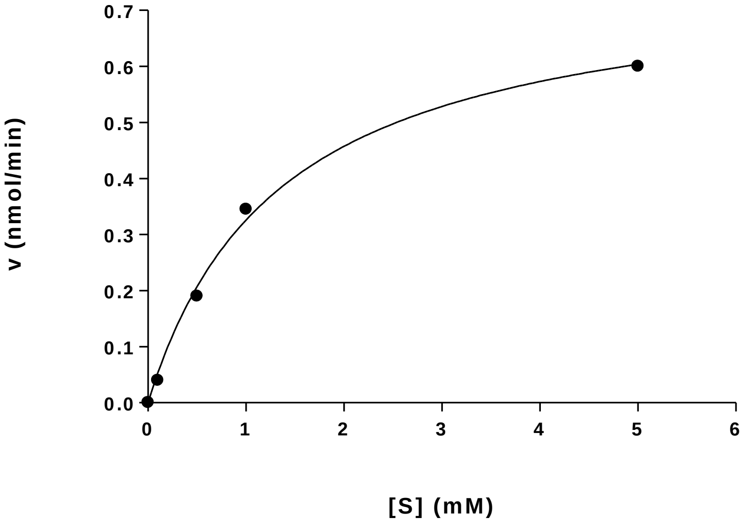

Michaelis-Menten plots of At5g36160. The enzyme assays used to draw the Michaelis-Menten plots are described in the "materials and methods" section. V_{max} is represented in nmoles/min , the K_m of the various substrates is represented in millimolar (mM). The measurements were made under optimal conditions with respect to pH, temperature and substrate concentrations. The kinetic constants were calculated by non-linear regression analysis using the GraphPad Prism Version 3.03 software.

**2-ketoglutarate**	10µg recombinant enzyme<br>5 mM L-Phenylalanine
**L-Tyrosine**	10µg recombinant enzyme<br>20mM 2-ketoglutarate
**L-Phenylalanine**	10µg recombinant enzyme<br>20mM 2-ketoglutarate
**4-hydroxyphenylpyruvate**	20µg recombinant enzyme<br>0.3mM thio-NAD<br>0.3mM Coenzyme A<br>5mM L-Glutamate<br>200µg recombinant 2-ketoglutarate dehydrogenase
**Phenylpyruvate**	20µg recombinant enzyme<br>0.3mM thio-NAD<br>0.3mM Coenzyme A<br>5mM L-Glutamate<br>200µg recombinant 2-ketoglutarate dehydrogenase
**L-Glutamate**	20µg recombinant enzyme<br>0.3mM thio-NAD<br>0.3mM Coenzyme A<br>5mM 4-hydroxyphenylpyruvate<br>200µg recombinant 2-ketoglutarate dehydrogenase

Note: the assays were in 500µl final volume using 100mM HEPES pH (8.2)
